# Emerging Roles of T Cells in the Pathogenesis of Nonalcoholic Steatohepatitis and Hepatocellular Carcinoma

**DOI:** 10.3389/fendo.2021.760860

**Published:** 2021-10-28

**Authors:** Petra Hirsova, Adebowale O. Bamidele, Haiguang Wang, Davide Povero, Xavier S. Revelo

**Affiliations:** ^1^ Division of Gastroenterology and Hepatology, Mayo Clinic, Rochester, MN, United States; ^2^ Department of Integrative Biology and Physiology, University of Minnesota, Minneapolis, MN, United States; ^3^ Department of Biochemistry and Molecular Biology, Mayo Clinic, Rochester, MN, United States; ^4^ Center for Immunology, University of Minnesota, Minneapolis, MN, United States

**Keywords:** T cells, NASH, HCC, Inflammation, CD4/CD8 lymphocytes

## Abstract

Nonalcoholic fatty liver disease (NAFLD) has become the most common chronic liver disease worldwide. A significant proportion of patients with NAFLD develop a progressive inflammatory condition termed nonalcoholic steatohepatitis (NASH), which may eventually advance to cirrhosis and hepatocellular carcinoma (HCC). NASH is characterized by steatosis, hepatocyte ballooning, and lobular inflammation. Heightened immune cell infiltration is a hallmark of NASH, yet the mechanisms whereby hepatic inflammation occurs in NASH and how it contributes to disease initiation and progression remain incompletely understood. Emerging evidence indicates that intrahepatic T cell immune mechanisms play an integral role in the pathogenesis of NASH and its transition to HCC. In this review, we summarize the current knowledge regarding the T cell-mediated mechanisms of inflammation in NASH. We highlight recent preclinical and human studies implicating various subsets of conventional and innate-like T cells in the onset and progression of NASH and HCC. Finally, we discuss the potential therapeutic strategies targeting T cell-mediated responses for the treatment of NASH.

## Introduction

The growing prevalence of obesity and type 2 diabetes worldwide is driving a proportional increase in the incidence of nonalcoholic fatty liver disease (NAFLD), a condition in which the liver accumulates excess fat unrelated to excessive alcohol consumption or secondary causes such as medication. NAFLD is strongly associated with risk factors such as obesity, type 2 diabetes, dyslipidemia, and hypertension, and is often considered the hepatic manifestation of the metabolic syndrome (1, 2). NAFLD affects approximately 25% of the world population and represents the most common chronic liver impairment, contributing to the burden of end-stage liver disease worldwide (3).

**Figure 1:**
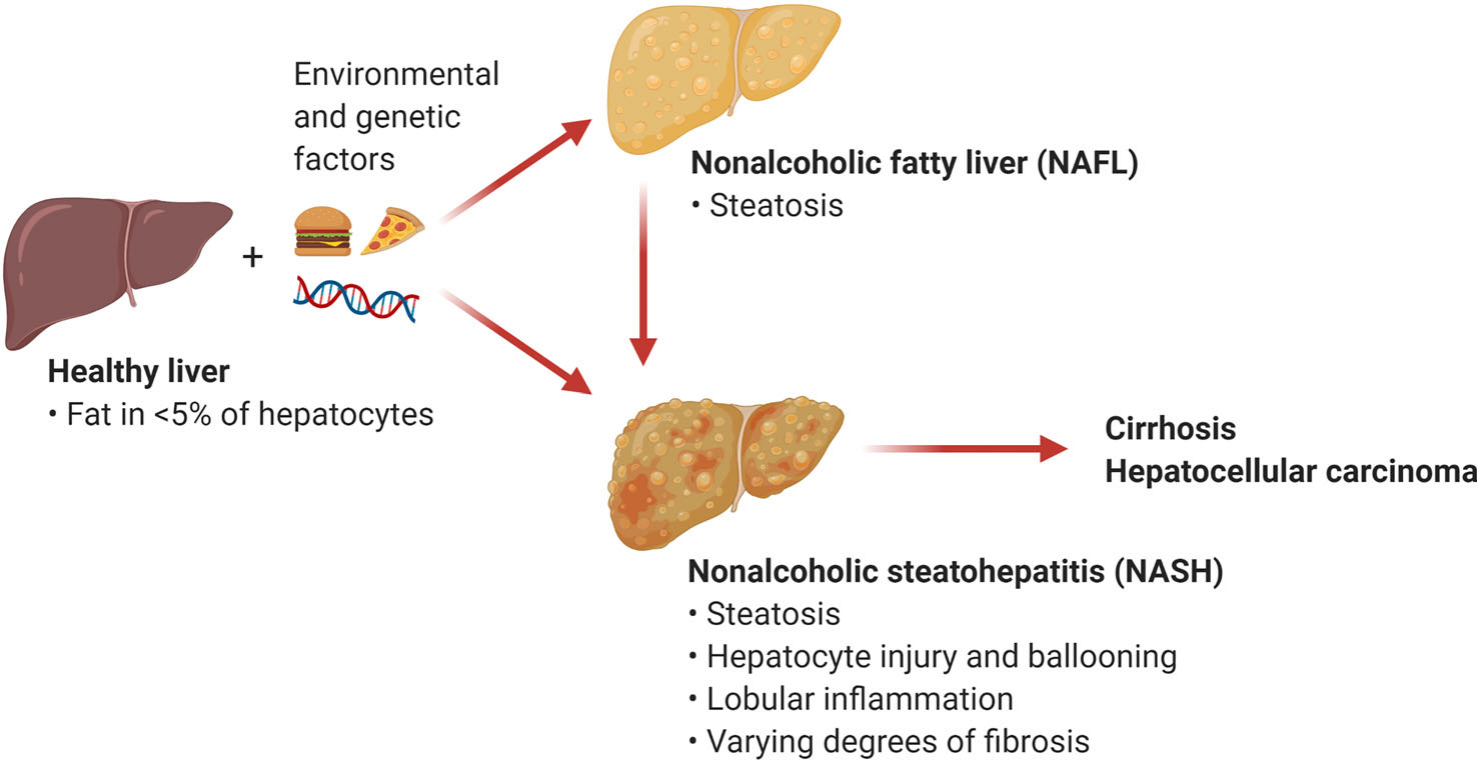
Nonalcoholic fatty liver disease (NAFLD). NAFLD encompasses a spectrum of hepatic manifestations such as nonalcoholic fatty liver (NAFL) and nonalcoholic steatohepatitis (NASH) that develop on the background of genetic and environmental factors including excess calories and sedentary lifestyle. NASH is a progressive condition that can advance to cirrhosis and hepatocellular carcinoma. (Created with BioRender.com).

The umbrella term NAFLD comprises a wide array of manifestations ranging from relatively benign conditions such as fatty liver to more severe disorders including nonalcoholic steatohepatitis (NASH) and NASH-induced cirrhosis and hepatocellular carcinoma (HCC). Nonalcoholic fatty liver (NAFL), also known as steatosis, is characterized by the accumulation of triglycerides in large lipid droplets within the hepatocytes in the absence of liver inflammation (**Figure 1**). A more advanced condition termed NASH is characterized by liver steatosis, hepatocellular injury and ballooning, and lobular inflammation. Chronic liver inflammation in NASH promotes tissue remodeling and fibrogenesis, which manifests as perisinusoidal fibrosis that can progress to bridging fibrosis and cirrhosis. Notably, the presence of liver fibrosis is the main predictor of morbidity and mortality in patients with NASH (2). An estimated 20% of patients with NASH progress to cirrhosis over two to four decades (2, 4). NASH also increases the risk for the development of HCC independently of cirrhosis (5). Current treatment options for NASH are limited to weight loss and lifestyle modification (6). There are no currently approved therapies for the treatment of NASH, although several drugs and their combinations are at various stages of clinical development with suboptimal preliminary results (7). Since inflammation plays an integral role in the transition from NAFL to NASH, understanding the mechanisms triggering hepatic inflammation during NASH and its progression to fibrosis and hepatocarcinogenesis could guide the development of new therapies. Here, we review recent evidence supporting the role of T cell subsets and T cell-mediated responses in the pathogenesis of NASH and NASH-induced HCC. We focus on the mechanisms of activation and effector function of intrahepatic conventional and innate-like T cells. In addition, we discuss opportunities for targeting these pathways as potential NASH therapies.

## Immune Mechanisms of Inflammation on NASH

**Figure 2:**
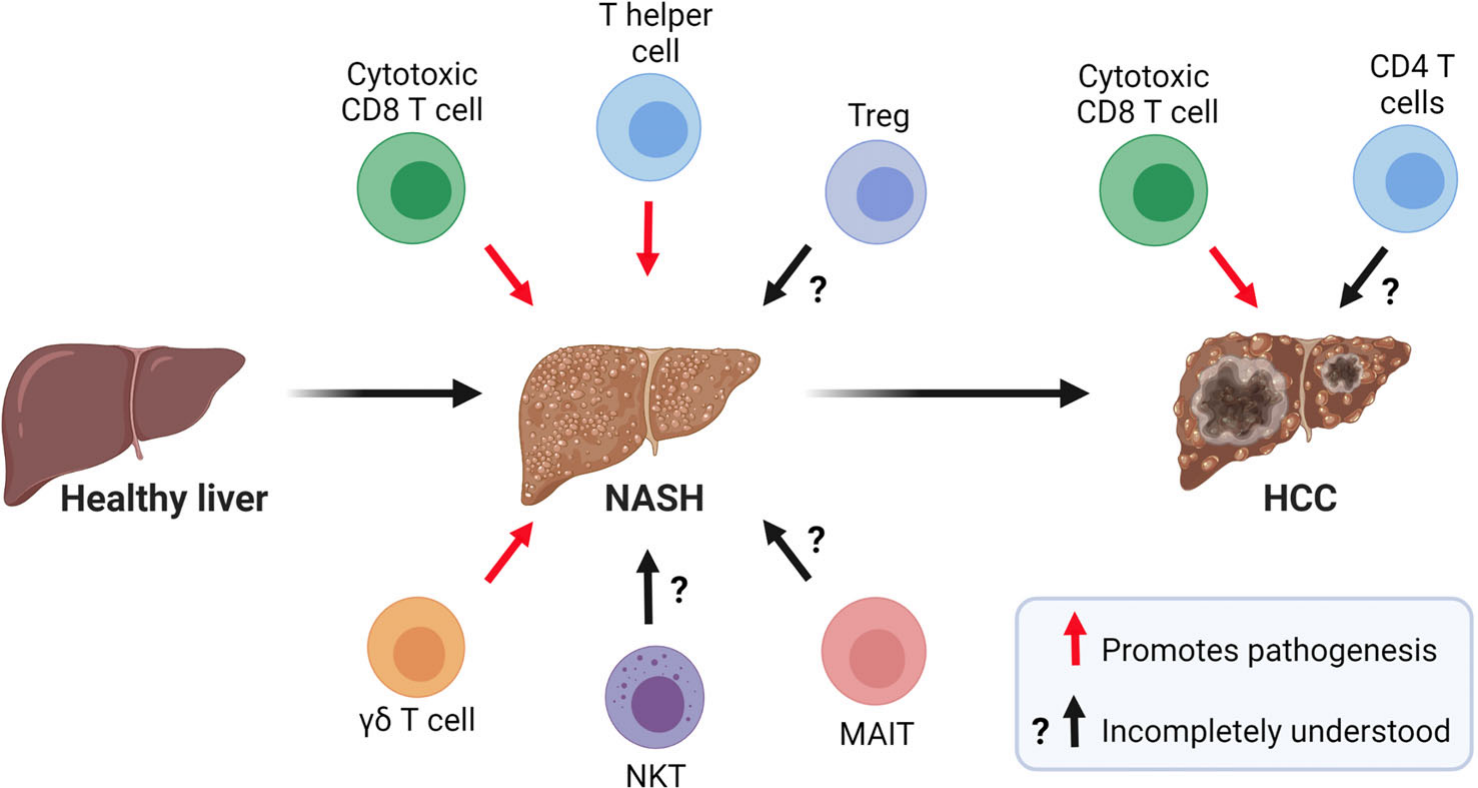
Role of T cells in the pathogenesis of NASH and NASH-related HCC. Current evidence suggests that CD8 T cells, CD4 T helper cells, and γδ T cells contribute to NASH pathogenesis. The role of NKT cells, MAIT cells, and Tregs in NASH is incompletely understood. In NASH-related HCC, a subset of activated and exhausted CD8 T cells promote tumorigenesis while the role of CD4 T cells in this process warrants further investigation. (Created with BioRender.com).

Growing evidence indicates that innate and adaptive immune mechanisms are critical components of steatosis progression, insulin resistance, inflammation, and fibrosis during NASH (8). The healthy liver is a unique immunological site containing a large number of immune cells that tolerate harmless foreign antigens and maintain tissue homeostasis (9). In contrast, NASH is characterized by a substantial accumulation of innate and adaptive immune cells that become activated and promote inflammation (10) (**Figure 2**). Innate immune cells, particularly macrophages, have emerged as essential players in the progression of NASH and have been the focus of intensive research (11). In homeostatic conditions, the liver contains a population of tissue-resident macrophages known as Kupffer cells (KCs) that originate from yolk sac progenitors and are scarcely replaced by monocytes from hematopoietic origin (12). In the liver, the maintenance of KCs is shaped by the production of macrophage growth factors by neighboring cells, most notably hepatic stellate cells (HSCs) (13). During the initiation of NASH, KCs sense disturbances in the tissue, such as increased lipopolysaccharide and fatty acids, and initiate hepatic inflammation and fibrosis through the production of tumor necrosing factor (TNF)α (14) and interleukin (IL)-1β (15). KCs can also release chemokines that promote the infiltration of proinflammatory monocyte-derived macrophages (MoMFs) (16). Single-cell RNA sequencing studies have recently shown that NASH not only promotes an increased infiltration of MoMFs, but also a loss of embryonically derived KCs that are partially replenished by monocyte-derived KCs (17, 18). Although, the accumulation of macrophages during NASH is associated with the progression of the disease, a remarkable subset of macrophages expressing the lipid receptor Trem2 has been shown to promote tissue homeostasis by coordinating hepatocyte energy supply and mitochondrial function (19). In addition to macrophages, the liver contains a population of dendritic cells (DCs) comprised of diverse subsets of classical (cDCs) and plasmacytoid DCs that reside in the subcapsular space, between hepatocytes, and in the vasculature (20, 21). The role of DCs in NASH is less clear with conflicting results likely reflecting their heterogeneity and differences in mouse models of disease. For instance, studies using mouse models of NASH have proposed a role for cDCs in preventing fibrosis and inflammation (22, 23). In contrast, recent single-cell analysis has revealed that type 1 cDCs are conserved immunological instigators of NASH in mice and humans (24). The evidence for a role of neutrophils is less controversial as these cells have been reported to promote NASH through the secretion of elastase (25) and myeloperoxidase (26).

Emerging evidence has implicated adaptive immunity involving T and B cells in the triggering and progression of NASH (27). Although limited, several studies using mouse models and observational studies in humans suggest that B cells have a pathogenic function in the progression of NASH (28). Indeed, patients with NASH show an increased number of intrahepatic B cells within T cell-rich aggregates and higher levels of circulatory IgG against oxidative stress-derived epitopes (29). Depletion of B2 cells in mice fed a western (30) or a methionine- and choline-deficient (MCD) (29) diet prevents hepatic inflammation and reduces T helper (Th)1 responses, suggesting a causative role for B cells in NASH progression. Mechanistically, NASH livers seem to accumulate B cells with elevated proinflammatory cytokine secretion and antigen-presentation ability following activation through the innate adaptor myeloid differentiation primary response protein 88 (28). Future research is needed to provide mechanistic insights into whether intrahepatic B cells are causative or consequential to NASH pathogenesis and their potential as key inducers of hepatic fibrosis.

**Figure 3:**
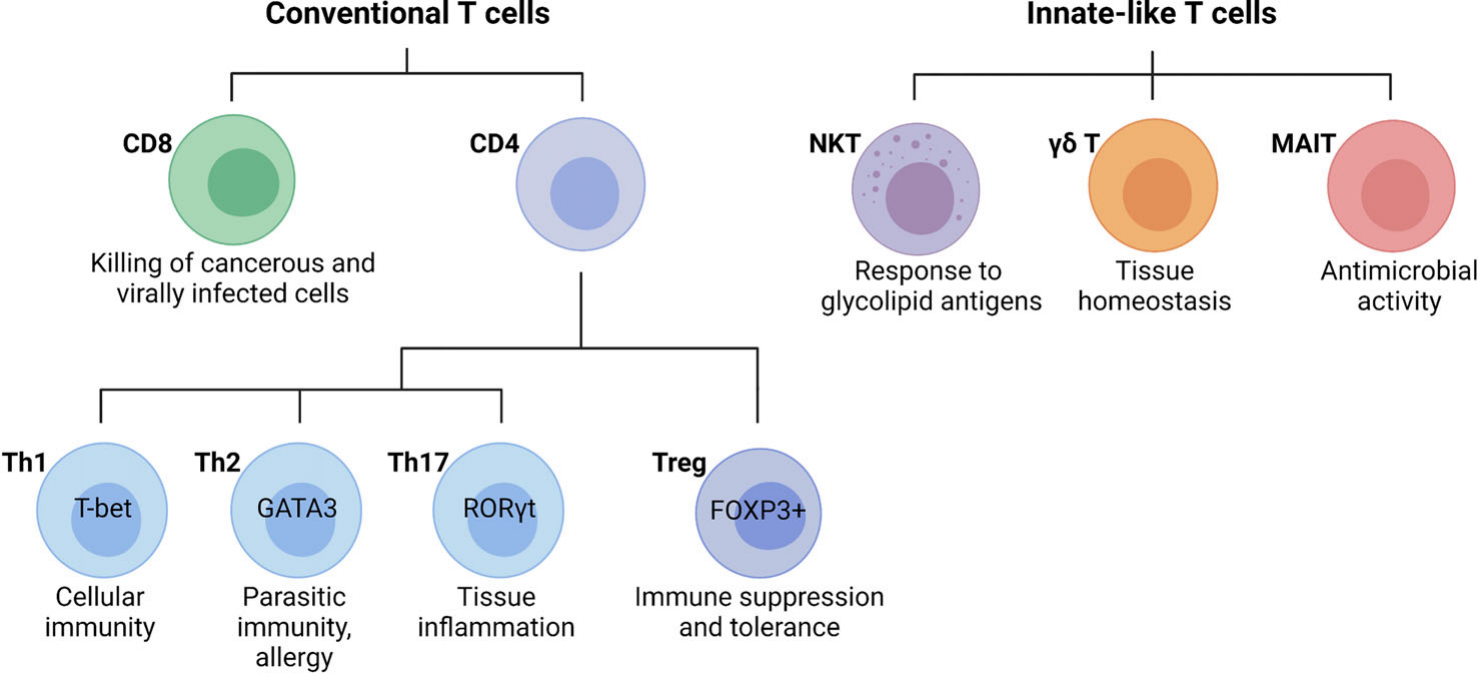
Main T cell subsets and their functions. Conventional T cells are broadly grouped into CD8 and CD4 T cell subsets with diverse functions during an immune response. In general, CD8 cytotoxic T cells directly kill cancerous or infected cells, while CD4 T cells regulate the immune response to a particular antigen. CD4 T cells can be classified as T helper (Th) cells or regulatory T cells (Tregs). Differentiated CD4 T cell subsets are marked by the expression of the lineage-specific transcription factors T-bet (Th1), GATA3 (Th2), RORγt (Th17), and FOXP3 (Tregs), which are critical for their differentiation and function. Innate-like T cells are a highly diverse group of cells including natural killer T (NKT) cells, gamma delta (γδ) T cells, or mucosal-associated invariant T (MAIT) cells. (Created with BioRender.com).

T cells are lymphocytes that mature in the thymus and recognize antigen using a cell surface, highly variable T cell receptor (TCR). Conventional T cells can be broadly classified into CD8 and CD4 T cells, based on the expression of glycoproteins that serve as TCR co-receptors (**Figure 3**). Functionally, T cells can be divided into cytotoxic CD8 T cells, CD4 T helper cells, and CD4 regulatory T cells (31). CD8 T cells are important for killing cancerous or virally infected cells as they recognize antigen presented by major histocompatibility complex (MHC)-class-I molecules. Upon activation, naïve CD8 T cells differentiate into effector cells with cytolytic and cytokine-producing capacity or memory cells that provide an enhanced response when the same antigen is encountered again (32). Memory CD8 T cells were originally subclassified as either effector (rapid response) or central memory (slower response). However, a third population known as tissue-resident memory T (TRM) cells that mainly reside in non-lymphoid tissues and are ready to mount an immediate immune response has been recently identified (33). Naïve CD4 T cells develop in the thymus and are exported to peripheral lymphoid sites following positive selection of double-positive thymocytes expressing functional αβ TCRs that bind MHC-class-II molecules (34). Upon activation, clonal expansion, and differentiation into specific subsets, CD4 T cells play a major role in immune responses and tissue homeostasis (35). Naïve CD4 T cells (CD62L+ in mice and CD45RA+ in humans) become activated through TCR-mediated recognition of antigenic peptides presented by MHC-class-II-expressing antigen-presenting cells such as DCs that provide obligatory co-stimulatory signals including CD28 ligation (35). Depending primarily on the cytokine milieu and metabolic cues provided by the microenvironment, CD4 T cells differentiate into effector T helper (Th) cell subsets including Th1, Th2, and Th17 cells, or regulatory T cells (Tregs, **Figure 3**) (36). Effector T helper cells are generally short-lived, although a proportion of them can differentiate into memory T cells that participate in long-term immunity or rapid recall immune response (35). Tregs are important for self-tolerance and suppressing autoimmune and inflammatory reactions as evidenced by multi-organ autoimmune disease observed in humans and mice lacking functional forkhead box protein P3 (FOXP3) (37, 38). Besides the classical T cell subsets, there are several populations of innate-like T cells such as the natural killer T (NKT), gamma delta (γδ) T cells, or mucosal-associated invariant T (MAIT) cells. Invariant NKT (iNKT) cells exist in an effector state and, upon activation, can rapidly produce a variety of cytokines that can determine the milieu for subsequent immune responses (39, 40). Unlike most T cells, γδ T cells are enriched in peripheral tissues such as the intestines, lungs, and skin where they contribute to immune responses. Although their functions are still being uncovered, γδ T cells have been shown to play roles in homeostasis, including the regulation of adipose tissue thermogenesis, tissue repair, and synaptic plasticity in the central nervous system (41). MAIT cells are found primarily in the blood, lungs, liver, and mucosa where they participate in the defense against bacterial and viral infections. This review will focus on the specific roles of the T cell subsets in the liver during the pathogenesis of NASH.

## Cytotoxic CD8 T Cells

At steady-state, the liver contains a distinct population of non-recirculating CD8 TRM cells that present an activated phenotype characterized by the expression of CD69 and are local immune sentinels (42). During inflammation or immune responses to pathogens, hepatic CD8 TRM cells can acquire an effector memory phenotype (CCR7- CD44+ CD62L-) that secretes interferon (IFN)γ or a central memory phenotype (CD44+ CCR7+ CD62L+) that is important for long-term immunity (43). This classification is based on the principle that effector memory T cells home to peripheral tissues while central memory T cells require CCR7 and CD62L to circulate between blood and secondary lymphoid tissues (44). Although CD8 T cell effector and memory subsets show a remarkable diversity of systemic and tissue-specific functions during immune responses to pathogens, the precise role of hepatic CD8 T cells in a chronic sterile inflammatory disease such as NASH remains incompletely understood. Recent advances in single-cell technologies, however, have provided mechanistic insights into the mechanisms of activation and effector functions of CD8 T cells in NASH.

### Role of Cytotoxic CD8 T Cells in NASH

Growing evidence shows that CD8 T cells accumulate in the liver of patients with NASH (45–48) and mouse models of the disease (46, 49). In patients with NASH, the increased number CD8 T cells in the liver correlates with elevated frequency of blood CD8 T cells expressing perforin, IFNγ, and TNFα, raising the possibility of systemic activation or crosstalk with other tissues (50). Experimental depletion of total CD8 T cells in animal models ameliorates NASH (51) and subsequent transition to HCC (46), suggesting that CD8 T cells directly instigate disease progression. Indeed, improved NASH in CD8 T cell-deficient mice is accompanied by restored hepatic insulin sensitivity (52), decreased liver damage (46), and reduced fibrosis (49). Although not completely understood, recent studies have revealed several mechanisms by which CD8 T cells become activated leading to NASH initiation or progression. Early during the transition from steatosis to NASH, activation of cytotoxic CD8 T cells is supported by type I interferons, resulting in increased production of proinflammatory cytokines IFNγ and TNFα (52). In addition, intrahepatic CD8 T cells from obese-hyperlipidemic mice with NASH express increased expression of IL-10 and show an ability to directly activate HSCs *ex vivo* (47). Although the existing evidence is consistent with the notion that total CD8 T cells are pathogenic in the development of NASH, the use of single-cell genomics reveals functionally distinct subsets of CD8 T cells. In healthy human livers, the majority of CD8 T cells are conventional αβ TCR+ T cells with enriched expression of CD69 and a tissue-resident memory phenotype (42). During established NASH, a subset of CXCR6+ CD8 T cells accumulate in the liver of patients and mice where they show features of short-lived tissue-resident effector cells. Functionally, CXCR6+ CD8 T cells express granzyme, TNFα, IFNγ, and programmed cell death protein 1 (PD-1), suggesting an activated exhausted phenotype during NASH. In a sequential process triggered by IL-15, metabolic signals, such as acetate and extracellular ATP activate CXCR6+ CD8 T cells that promote non-specific killing of hepatocytes and instigates disease progression (45). Importantly, the progressive accumulation of exhausted PD-1+ CD8 T cells during NASH is not limited to causing tissue damage but can lead to impaired immune surveillance and the development of NASH-driven HCC (48). Despite the growing evidence supporting a pathogenic role for CD8 T cells in NASH through their production of cytokines and auto-aggressive ability, a protective role for CD8 T cell-derived perforin in preventing hepatic inflammation in NASH has been proposed in mice (53). In addition, recent evidence suggests that CD8 T cells are required for the resolution of inflammation and fibrosis in a mouse model of NASH resolution driven by a switch from a NASH-inducing to a normal chow diet that fully reverses the disease (54). During the resolution phase in this model, CD8 T cells show a tissue-resident memory phenotype and can inhibit liver inflammation and fibrosis upon transfer into NASH mice (54). Overall, evidence from human and mouse studies suggests that CD8 T cells instigate NASH progression through secretion of proinflammatory molecules and non-specific killing of hepatocytes. However, CD8 T cells may use specific effector functions, such as perforin, to limit inflammation during NASH. Intriguingly, tissue-resident CD8 T cells are required for the resolution of murine NASH suggesting a potential dichotomous role during disease progression and resolution.

## CD4 T Cells

The balance between the functions of Th effector and Treg CD4 T cell subsets plays an important role in maintaining tissue homeostasis. While Th cells can instigate immune responses against pathogens, allergens, and tumors, Tregs promote immune tolerance and facilitate tissue repair and wound healing. Indeed, loss of the balance between T cell effector and regulatory functions can lead to autoimmune and inflammatory disease. CD4 T cell subsets can be identified by the expression of subset-specific transcription factors (T-bet for Th1; GATA3 for Th2; RORγt for Th17; FOXP3 for Tregs) (36). T cell lineage-defining transcription factors, cytokine signaling, and metabolic and epigenetic cues orchestrate the development and function of CD4 T cells. Furthermore, CD4 T cell subsets exhibit some degree of heterogeneity and plasticity between phenotypes (55). Classical Th and Treg cell subsets are characterized by the secretion of specific cytokines that can influence immune and non-immune cells (36). Th1 cells mainly secrete IFNγ, IL-2, TNFα, and lymphotoxin-α and are involved in eliminating intracellular pathogens and tumor cells (56). Th1 differentiation is mainly driven by IL-12 and IFNγ produced by DCs (56). Th2 cells orchestrate protective type 2 immune responses against helminth parasites through the secretion of IL-4, IL-5, and IL-13, which are also involved in asthma and allergic disease (56). Immune cell-derived IL-4 and epithelial-derived IL-25 and IL-33 regulate Th2 cell development (56). Th17 cells produce IL-17A, IL-17F, and IL-21, and their development is governed by IL-6, TGF-β, IL-1β, and IL-23 (57). Although Th17 cells show protective functions against bacteria and fungi infections, excessive release of Th17 cytokines has been implicated in chronic inflammation (58). Tregs express FOXP3 and CD25 (IL-2 receptor alpha) and can be classified into natural Tregs, that derive from the thymus *via* TCR-mediated recognition of self-antigens, and Tregs induced in the periphery in the presence of IL-2 and TGF-β (59). Treg instability, plasticity, or transdifferentiation have been reported to regulate tissue homeostasis and mediate intestinal inflammation (60–62). Overall, a precise understanding of the molecular mechanisms that regulate the differentiation and effector or regulatory functions of CD4 T cells will have implications in the treatment of infections and immune-mediated diseases.

### Role of CD4 T Cells in NASH

Dysregulation of CD4 T cell function is emerging as a factor involved in NAFLD and NASH progression. Profiling of peripheral and intrahepatic leukocytes has revealed an increase in the accumulation of inflammatory Th1 and Th17 cells during NASH in humans and mouse models of disease (27, 63, 64). To better understand human-relevant T cell responses during NASH progression, intrahepatic T cells were characterized in a humanized mouse model in which irradiated NOD-*scid IL2rg* ^null^ mice were engrafted with human immune cells and fed a high-fat, high-carbohydrate (HFHC) diet (64). These humanized mice displayed a progressive increase in the frequency of human peripheral central memory and effector memory CD4 T cells during HFHC diet feeding. More importantly, antibody-mediated depletion of total CD4 T cells reduced the amounts of hepatic inflammatory cytokines, NAFLD activity score, and fibrosis in these humanized NASH mice, suggesting that disease development is a CD4 T cell-dependent process (64). Supporting a potential role for CD4 T cells in promoting NASH through the release of proinflammatory cytokines, whole-body IFNγ deficiency attenuates disease progression in mice fed an MCD-high-fat diet including a reduced activation and infiltration of intrahepatic macrophages (65). In agreement, a higher frequency of CD4 T cells expressing IFNγ and IL-4 has been reported in the peripheral blood of patients with NASH compared with healthy individuals (63). Although the mechanisms responsible for the expansion of CD4 T cells in NASH are unclear, recent evidence suggests that their recruitment into the liver through an integrin-ligand interaction is an important step in NASH progression. CD4 T cell recruitment into inflamed tissues involves the interaction between the T cell integrin α4β7 and its ligand, the mucosal addressin cell adhesion molecule 1 (MAdCAM-1) expressed on the liver endothelium and colonic mucosa (66). Mice with NASH, induced by genetic deletion of the junctional adhesion molecule A, show an increased number of peripheral, intestinal, and intrahepatic CD4 T cells expressing the integrin α4β7 (67). Importantly, blockade of α4β7 or MAdCAM-1 reduces the recruitment of CD4 T cells into the intestine and NASH liver and attenuates hepatic inflammation and fibrosis (67). Thus, these data emphasize the role of intestinal barrier disruption in CD4 T cell-mediated NASH pathogenesis through α4β7-MAdCAM−1 interaction. Despite the evidence showing that the accumulation of Th1 and Th17 cells promotes NASH, other studies suggest that dysregulation of lipid metabolism during NAFL and NASH causes a selective loss of CD4 T cells that leads to impaired anti-tumor surveillance in mouse models and human samples (68, 69). Future research is needed to clarify the factors that trigger the accumulation of proinflammatory Th1 cells and promote NASH progression and reconcile them with the data showing a loss of CD4 T cells during disease.

An increased Th17 cell accumulation has been observed in the liver of patients with NASH, relative to those with NAFL, suggesting that intrahepatic Th17 cells or IL-17 may not only promote disease progression but also help discriminate between NAFL and NASH (63). Indeed, inhibition of IL-17 and its receptor protected mice against NASH induced by a high-fat or MCD diet (70–72). Despite the important function of Tregs in preventing the excessive activation of pathogenic immune cells, very few studies have focused on the specific role of Tregs during NASH progression. A lower frequency of resting Tregs and a higher percentage of IFNγ- and IL-4-expressing Tregs were observed in the peripheral blood of patients with NAFL and NASH (63). Similarly, the liver of patients with NASH displayed a trend towards a lower frequency of resting Tregs and an upward trend in activated Tregs compared to NAFL patients (63). Mice with NASH induced by feeding a high-fat diet and challenged with endotoxin showed a depletion of Tregs in the liver while their adoptive transfer attenuated liver injury and inflammation, suggesting a direct role for Tregs in preventing disease progression (73). However, a recent report showed that Tregs increase in the liver during NASH, and that their depletion inhibits the progression from NASH to HCC in a mouse model of choline-deficient, high-fat diet feeding and diethylnitrosamine injection (74). These conflicting findings regarding the role of Tregs in NASH could be explained by the different mouse models of disease used in these studies or the possibility that Tregs have opposite functions in early and late NASH with the subsequent carcinogenesis.

The mechanisms underlying the activation of CD4 T cells and subsequent effector functions in NASH are largely underexplored. However, the liver microenvironment and cellular metabolism may govern the acquisition of distinct phenotypes by CD4 T cell subsets. For example, the inflammatory and highly glycolytic CXCR3+ Th17 (ihTh17) cells have been recently implicated in obesity and NAFLD pathogenesis (75). In contrast to conventional Th17 cells, ihTh17 cells display increased chromatin accessibility as well as a transcriptomic profile indicative of enhanced glycolytic capacity, CXCR3 activation, and production of inflammatory IFNγ, TNFα, and IL-17A (75). As a result, glycolysis inhibition, ihTh17 cell-specific ablation of IFNγ, or modulation of CXCR3-CXCL9/10 axis conferred protection against hepatocellular damage and hepatic accumulation of ihTh17 cells in murine models of NASH (75). These findings highlight how the liver microenvironment and metabolism can dictate the function of Th17 cells during NAFLD pathogenesis. Additional studies are needed to identify the triggers of CD4 T cell activation and differentiation into Th1 and Th17 subsets that promote liver inflammation during NASH.

## Innate-Like T Cells

Innate-like T lymphocytes are a diverse group of immune cells that share features of both innate and adaptive immune systems and do not fit into the traditional innate or adaptive classification. Hepatic innate-like T lymphocytes, such as natural killer T (NKT) cells, γδ T cells, and mucosal-associated invariant T cells (MAITs), have been implicated in the pathogenesis of NASH in humans and mice (76).

### Natural Killer T Cells

Invariant natural killer T cells (iNKT) express a semi-variant TCR, which is composed of Vα14-Jα18 and Vβ2, Vβ7, or Vβ8.2 in mice or Vα24-Jα18 coupled with Vβ11 in humans (77, 78). Unlike αβ T cells that recognize peptide antigens, iNKT cells exclusively recognize lipid antigens presented by CD1d molecules (77, 78), allowing their detection using a CD1d tetramer loaded with cognate lipid antigen (79, 80). iNKT cells are widely distributed throughout tissues but only represent a relatively small population (0.1% - 1%) of total immune cells (39). In the steady-state, iNKT cells are highly abundant in the liver, accounting for up to 30% of total intrahepatic lymphocytes in mice (39) and 3 - 5% of T cells in humans (81). Because of this property, iNKT cells are identified as innate-like T cells along with other specialized T cell subsets, such as MAIT cells (82). The majority (75 - 80%) of iNKT cells in the liver express the chemokine receptor CXCR6, which is required for their local enrichment and retention (83). Intravital fluorescence microscopy imaging of CXCR6-reporter mice has shown that iNKT cells are motile and actively patrol liver sinusoids, suggesting that iNKT participate in the intravascular immune surveillance in the liver (83).

#### Role of Cytotoxic iNKT Cells in NASH

Several studies have reported that the number of hepatic iNKT cells decreases in steatotic livers in mouse models of NAFL (84–87). Such loss of iNKT cells has been attributed to increased apoptosis induced by IL-12 derived from Kupffer cells (88) and enhanced Tim-3/Gal-9 signaling (89). Supporting a direct role of iNKT cells in NAFLD, mice lacking iNKT cells show higher susceptibility to developing fatty liver, enhanced weight gain, and insulin resistance following high-fat diet feeding, while adoptive transfer or activation of iNKT cells with a cognate lipid antigen (αGalCer) reverses this phenotype (84). The protective role of iNKT cells against fatty liver disease and metabolic disturbances seems to persist during the transition from fatty liver to NASH (90, 91). Nonetheless, conflicting data have suggested a pathogenic role for iNKT cells in mouse models of NASH. For instance, iNKT cells have been shown to promote fibrosis during NASH using CD1d-deficient mice fed an MCD diet (92). Similarly, in db/db mice or animals fed a choline-deficient L-amino acid–defined diet, iNKT cell deficiency ameliorates tissue inflammation, hepatic steatosis, and fibrosis (87, 93). Moreover, a recent study using a choline-deficient, high-fat diet (CD-HFD) NASH mouse model showed that the number and frequency of iNKT cells did not change in NASH but Ja18- and CD1d-deficient mice that lack iNKT cells had more severe NASH comparable to wild-type controls (45). These contradictory findings can be attributed to the differences in mouse models used to promote NASH. In addition, iNKT subsets with diverse functions may have opposite roles in regulating disease progression. Despite being monospecific, iNKT cells are a heterogeneous population composed of multiple functionally distinct subsets such as NKT1, NKT2, NKT17, NKTFH, and NKT10, defined by the expression of key transcription factors and cytokine production (77, 78). Although most iNKT cells in the mouse liver are NKT1 cells that express T-bet and can secrete IFNγ following activation (39, 94), iNKT subsets can be prone to polarization and are plastic in adopting cell fates based on the environmental cues (95).

### Gamma Delta (γδ) T Cells

The ‘unconventional’ γδ T cells are T cells that express a distinct heterodimeric TCR composed of an γ chain and δ chain and are considered part of the innate immune system due to their innate-like characteristics. Unlike conventional αβ T cells, γδ T cells recognize a broad range of antigens without the presence of MHC molecules and thus can be activated without the help of antigen-presenting cells. Upon antigen recognition, γδ T-cell functional responses include cytotoxic activity on target cells, production of IL−17 and IFNγ, as well as activation of other immune cells, leading to pathogen clearance, tissue inflammation or homeostasis (96). Intrahepatic γδ T cells are estimated to represent 3–5% of murine and 8-15% of human lymphocytes in the liver, which is a several-fold higher frequency than in other tissues (97). In mice, the microbiota appears to control the number of hepatic-resident γδ T cells and their homeostasis in a lipid antigen-CD1d-dependent manner (98). Intrahepatic γδ T cells, similar to adipose tissue γδ T cells (99), are a significant source of proinflammatory cytokine IL-17, which can be rapidly secreted upon stimulation (98).

#### Role of Hepatic γδ T Cells in NASH

The number of intrahepatic γδ T cells and IL-17-producing γδ T cells are substantially increased after high-fat diet feeding in mice (52, 98). Despite no changes in obesity, TCR δ knockout (Tcrd-/-) mice that lack γδ T cells display reduced liver injury and lobular inflammation following high-fat or HFHC diet feeding, suggesting that γδ T cells promote NASH (98). IL-17 secretion by γδ T cells has been proposed to be the instigating factor as Tcrd-/- mice reconstituted with hepatic Il17a-deficient γδ T cells show ameliorated NASH, compared with those receiving WT γδ T cells (98). Similarly, Tcrd-/- mice fed an MCD diet displayed attenuated liver steatosis, injury, and hepatic leukocyte infiltration, including a decreased influx of inflammatory monocytes (100). In this model, however, the pathogenic role of γδ T cells was independent of IL-17, likely reflecting the disparities in disease pathogenesis induced by the different NASH-inducing diets. An outstanding question is whether γδ T cells influence the development of fibrosis. Nevertheless, the available data support the notion that intrahepatic γδ T cells contribute to the pathogenesis of NASH.

### Mucosal-Associated Invariant T Cells

MAIT cells are a unique T cell subset with innate-like features. MAIT cells express high levels of CD161 and an evolutionarily conserved semi-invariant TCR repertoire that recognizes microbial-derived non-peptide antigens presented by the MHC class-1 like molecule MR1. When activated by microbial antigens or cytokines, MAIT cells rapidly secrete proinflammatory factors, such as TNFα, IFNγ, and IL-17, or exert cytotoxic activity on target cells (101). In humans, MAIT cells are enriched in the liver, compared with peripheral blood, and reside in the portal tracts close to bile ducts (102). Interestingly, MAIT cells are substantially less abundant in mice than in humans in several tissues including the liver (97).

#### Role of Hepatic MAIT Cells in NASH

The frequency of circulating MAIT cells is decreased in several chronic inflammatory liver diseases including NAFLD (102–104). In patients with NASH, MAIT cells show an activated phenotype with increased cytotoxic activity but a decreased expression of TNFα and IFNγ (101, 103). In the liver of patients with NAFLD, the number of MAIT cells was found to be increased and correlated with the disease activity score (103). The role of MAIT cells in NASH has been investigated using mice deficient in the MHC-I-like molecule Mr1, which lack MAIT cells (103). Compared to wild-type controls, MCD-fed Mr1-deficient mice had increased liver injury, steatosis, and NAFLD activity score. Such aggravated NASH progression in Mr1-deficient mice was accompanied by increased frequency of proinflammatory macrophages and decreased frequency of alternatively activated macrophages. *In vitro* co-culture experiments showed that activated MAIT cells could directly promote monocyte/macrophage polarization towards the alternatively activated phenotype (103). Overall, this study suggests that MAIT cells prevent NASH progression through indirect effects on macrophage polarization. Notably, MAIT cells have been shown to promote proinflammatory responses and HSC activation with consequent liver fibrogenesis in chronic and autoimmune liver disease (104, 105). Future studies are needed to clarify whether MAIT cells may play a role in NASH-associated fibrogenesis.

## T Cells in NASH-Associated Hepatocellular Carcinoma

HCC accounts for 70-80% of liver cancers and is the fourth-leading cause of cancer-related mortality worldwide (106). In recent years, there has been a substantial increase in HCC as a consequence of NASH, which is becoming one of the fastest-growing causes of HCC (107). Indeed, NASH-driven HCC accounts for 10 to 34% of the known etiologies of liver cancer (108). Although the pathophysiology of NASH-driven HCC is poorly understood, obesity and insulin resistance can promote chronic inflammation, dysregulation of lipid metabolism, and establishment of the carcinogenic microenvironment preceding HCC (109). Notably, NASH-driven HCC affects older individuals regardless of cirrhosis (5, 110). Over the past few years, a key role for adaptive immune cells in NASH-driven HCC development has emerged. Several reports have described increased recruitment of T cells in murine and human NASH-HCC and have revealed several mechanisms by which T cells regulate HCC development. As the role of immune cells in HCC has been recently reviewed elsewhere (111), here we focus on immune mechanisms that are specific to NASH as an underlying cause of HCC.

Activation of intrahepatic CD44- and CD69-expressing CD8 T cells has been reported to promote NASH and HCC through interactions with hepatocytes and by inducing liver damage in mice fed a CD-HFD (46). Under this feeding regimen, mice develop metabolic syndrome with liver steatosis, inflammation, fibrosis, and HCC. CD-HFD-fed Rag1-/- mice, which lack mature T and B cells, are completely protected against NASH and HCC development (46). Similarly, β2 microglubulin knockout (β2m-/-) mice that have a severe deficiency of CD8 T cells are protected from CD-HFD-induced liver damage and tumorigenesis, suggesting that CD8 T cells promote HCC (46). In a diet-induced NASH-HCC murine model, CD8 T cells not only lacked effective immune surveillance functions but also promoted HCC development (48). In humans, whole-exome sequencing of NASH-driven HCC liver shows an increased hepatic T cell accumulation and an enrichment of gene signatures associated with T lymphocytes (112). In this study, the cirrhotic NASH-driven HCC cases displayed common features of immune exhaustion, a state of dysfunction commonly associated with chronic T cell stimulation by tumor antigens (112). Mechanistically, it has been shown that inactivation of T cell protein tyrosine phosphatase (TCPTP) in hepatocytes can drive STAT-1 and STAT-3-dependent gene expression of T cell chemoattractants such as CXCL9 that promote T cell recruitment (113). However, the recruitment of T cells and subsequent NASH were not essential for the development of HCC during obesity. In contrast, inhibition of STAT-3 expression prevented HCC development but did ameliorate NASH and fibrosis (113). Despite the earlier evidence suggesting that they promote HCC, CD8 T cells were also shown to mediate immunosurveillance against NASH-HCC in high-fat diet-fed MUP-uPA mice that overexpress urokinase plasminogen activator in hepatocytes causing endoplasmic reticulum stress (114). In this study, IgA-producing cells were shown to interfere with the protective role of anti-tumor cytotoxic CD8 T cells leading to HCC. Notably, the ablation of CD8 T cells in IgA-deficient mice restored HCC development, suggesting that IgA-producing cells promote tumorigenesis directly through inhibition of CD8 T cells (114).

In a recent study, administration of anti-PD-1 immunotherapy to CD-HFD-fed mice increased the number of PD−1+ CD8 but not PD-1+ CD4 T cells, aggravated liver damage, and paradoxically augmented the incidence of liver cancer, independently of the degree of liver fibrosis (48). Consistently, the effects of PD-1 therapeutic blockade on liver cancer incidence were confirmed in a genetic model lacking PD-1 (48). Despite the large availability of cancer CD8 T cells upon therapeutic PD-1 or PD-L1-related immunotherapy, no signs of NASH-driven HCC regression were observed, demonstrating that PD-1+ CD8 T cell-mediated tissue damage leads to HCC development (48). Given that excessive hepatocyte apoptosis promotes HCC in NASH and CXCR6+ CD8 T cells cause non-specific hepatocyte killing (45, 115), CD8 T cells likely fueled the liver carcinogenesis in the CD-HFD model. Further investigation of NASH-derived CD8 T and PD-1+ CD8 T cells at single-cell transcriptomic level revealed that the latter expressed higher levels of markers of effector function (TNFα, IFNγ, granzyme), exhaustion (Eomes, PD-1, Ki67^low^), and liver residency (CXCR6, CD44) (48). Based on these findings, it has been postulated that anti-PD-1 immunotherapy leads to increased abundance specifically of liver resident PD-1+ CD8 T cells rather than extrahepatic T cell populations (48).

A loss of CD4 T cells but not CD8 T cells has been reported in the liver of NASH mice induced by the MCD diet and a choline-deficient L-amino acid-defined diet (69). Such loss of CD4 T cells was observed in both tumor-free and tumor-bearing environments, suggesting a tumor-independent regulation of intrahepatic T cells promoted by fatty liver. In turn, depletion of CD4 T cells in MCD diet-fed mice bearing MYC oncogene-driven HCC promotes hepatic carcinogenesis. Interestingly, CD4 T cells from MCD diet-fed mice were more susceptible to fatty acid accumulation, with a particular enrichment of linoleic acid uptake. *In vitro*, linoleic acid enhanced CD4 T cell, but not CD8 T cell, apoptosis due to incremented mitochondrial reactive oxygen species generation and activation of caspases (69). Together, these findings seem to indicate that hepatic T cell loss or exhaustion observed in this NASH model might compromise hepatic antitumor surveillance, thus increasing the susceptibility to NASH-induced transition to HCC development. Although hepatic CD4 T cells decline in NASH-HCC, the frequency of Th17 cells that support cancer development increase (114). Indeed, the up-regulation of the hepatic unconventional prefoldin RPB5 interactor (URI) by nutrient overload leads to DNA damage and NASH-HCC *via* the recruitment of Th17 cells and increased production of IL-17A (116). Importantly, blocking IL-17A signaling reduces liver injury and prevents HCC development (116). In contrast to CD8 T cells, depletion of CD4 T cells and anti-PD-1 immunotherapy failed to reduce the incidence of NAFLD-HCC (48). Interestingly, in mice that prophylactically received anti-PD-1 therapy along with CD-HFD, CD4 T cell immunodepletion reduced the tumor burden per liver and decreased the size of tumor nodules, suggesting CD4 T cell control tumor size. Furthermore, this study suggests that the depletion of CD4 T cells or regulatory T cells might contribute to tumor inhibition rather than development (48). The conflicting results regarding the role of CD4 T cells in experimental NASH-HCC models underscore the importance of the dietary interventions and emphasize the need for improved animal models of NASH-induced HCC that closely mimic human disease (117).

Taken together, these findings indicate that chronic liver inflammation sets the basis for the development of HCC. Recent studies suggest that anti-PD-1 treatment fails to restore exhausted PD-1+ CD8 T cells to execute anti-tumor surveillance and instead, anti-PD-1 promotes the expansion of this subset of CD8 T cells with enhanced HCC-inducing potential. As immunotherapy can reinvigorate antitumor immune responses to promote cancer regression, a careful stratification of HCC patients will be required to identify responders and non-responders in NASH-associated HCC

## Therapeutic Opportunities for Targeting T Cells in NASH

Given the role of T cells in the pathogenesis of NASH, it is conceivable that T-cell–directed therapies can prevent disease development. The possible approaches may include the depletion of specific pathogenic T cell subsets, inhibition of T cell recruitment into the liver, targeting of T cell-mediated cytotoxicity and proinflammatory factors, inhibition of T cell survival and proliferation, modulation of TCR signaling, and immunization. Several potential approaches have been tested in preclinical settings, including the inhibition of pathogenic CD4 T cell recruitment using a neutralizing monoclonal antibody against α4β7 that was successful at preventing binding of α4β7-bearing CD4 T cells to MAdCAM-1 in the liver and intestine and resulted in attenuated liver inflammation and fibrosis (67). Indeed, the anti-​α4β7 monoclonal antibody vedolizumab is approved for the treatment of inflammatory bowel disease, while an anti-MAdCAM-1 antibody is in clinical trials for the same indication. To target T cell pathogenic function, an improved understanding of the mechanisms instigating the pathogenic cytotoxic activity and effector functions is needed. Considering the evidence implicating the IL-17 axis in NAFLD and NASH, pharmacological inhibition of IL-17 signaling could be such a strategy as shown in a pre-clinical mouse model of alcohol-induced liver injury (118). In addition, targeting T cell metabolic programs may represent a therapeutic strategy to dampen their inflammatory function during NASH. For example, CD8 T cell auto-aggression toward hepatocytes is promoted by extracellular ATP, which can be prevented by using inhibitors of purinergic receptors or pannexin 1 (the ATP release channel) (45). Indeed, antagonists of purinergic receptors have been developed for clinical use and are currently being tested as a treatment for a variety of immune-mediated diseases (119). As NASH is characterized by the accumulation of exhausted CD8 T cells in the liver, reactivating exhausted CD8 T cells through manipulation of cellular metabolism such as restoring glycolytic pathways (120), holds the potential to reduce their inflammatory function and enhance their anti-tumor activity in NASH-driven HCC. Furthermore, clarification of the role of Tregs in NASH and HCC is needed as targeting Tregs to improve cancer immunotherapy and to treat autoimmune disease is being intensively evaluated (121). Finally, it is important to recognize that although T cell targeting therapies might ameliorate NASH progression, unwanted side effects may involve immunosuppression and toxicity. Therefore, targeting T cells will need to be carefully designed to specifically target pathogenic processes without interfering with T cell homeostatic functions.

## Concluding Remarks and Future Directions

**Figure 4:**
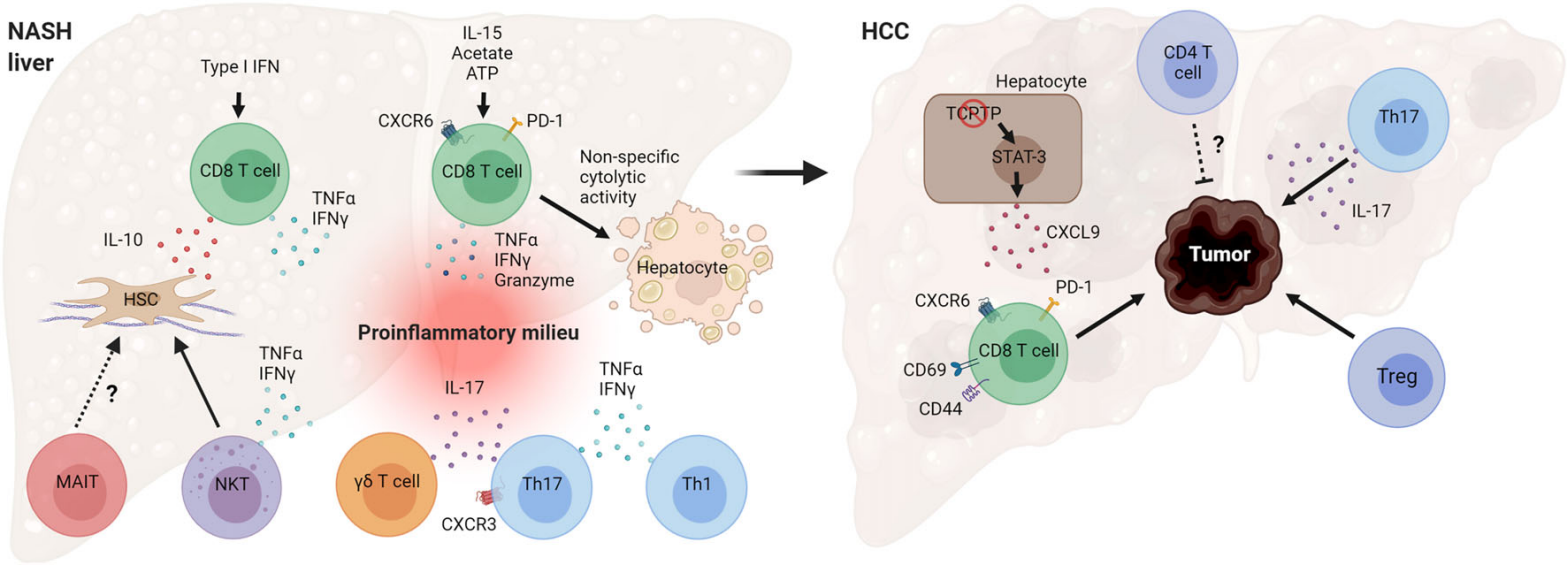
Mechanisms by which T cells can promote NASH and HCC pathogenesis. Hepatic CD8 T cells in NASH secrete cytokines that promote hepatic stellate cell (HSC) activation and tissue inflammation. NASH-associated CD8 T cells can also cause non-specific (antigen-independent) cell death of hepatocytes. T helper cells, Th17 and Th1, and γδ T cells secrete effector cytokines during NASH, contributing to inflammatory tissue milieu. Loss of T cell protein tyrosine phosphatase (TCPTP) in hepatocytes leads to STAT-3-dependent secretion of CXCL9 and liver tumorigenesis. Programmed cell death protein 1 (PD-1)+ CD8 T cells, Tregs, and cytokine IL-17 promote HCC development, while CD4 T cells overall may decrease tumor burden and control tumor size. (Created with BioRender.com).

NAFLD is a heterogeneous liver disease with a spectrum ranging from simple lipid accumulation to inflammation, liver injury, fibrosis, and HCC. Activation of the adaptive immune system during NASH is emerging as a key event in the pathogenesis of the disease. Substantial evidence, especially from preclinical research, indicates that several T cell subsets play a pathogenic role in NASH evolution (**Figure 4**). Cytotoxic CD8 T cells seem to promote liver injury and hepatocyte death in NASH, leading to NASH-related HCC. Recent work has revealed that the NASH liver accumulates a higher number of activated PD-1-expressing CD8 T cells that further expand following anti-PD1 treatment but fail to prevent HCC (48). Future work is needed to clarify how NASH increases the risk of HCC following anti-PD-1/PDL-1 therapy and the mechanisms by which activated CXCR6+ CD8 T cells promote carcinogenesis. CD4 T helper subsets, such as Th1 and Th17 cells, likely contribute to NASH pathogenesis through the secretion of effector cytokines. More studies are required to better understand the role of Tregs and innate-like T cells in the progression of NASH. There is also a substantial number of seemingly contradictory or variable results regarding the role of specific T cell subtypes. Several factors may contribute to these disparate findings, including differences in animal models used and heterogeneity of immune cell types. NASH preclinical research employs a great variety of mouse models, which differ significantly in the mechanisms by which they drive the disease pathogenesis (122). Certain models such as the MCD diet cause severe fibrotic NASH phenotype in the absence of obesity, adipose tissue inflammation, and insulin resistance. In contrast, NASH models involving western diet feeding cause obesity, adipose tissue inflammation, and insulin resistance and, therefore, are expected to have distinct metabolic and immune perturbations. The use of animal models with high fidelity to human etiology and pathogenesis is key for obtaining relevant insights into human disease pathogenesis.

Although identifying the factors that trigger inflammation in the transition from NAFL to NASH is a primary goal of the field (123), limited research has been conducted to determine the intrinsic and extrinsic mechanisms of T cell activation. So far, activation of CD8 T cells during NAFL and NASH has been attributed to type I interferons (52), IL-15, and metabolic signals such as acetate and extracellular ATP (45). In Th17 cells, modulation of cell-intrinsic glycolysis has been shown to decrease their inflammatory function during NAFLD (75), highlighting the need to determine how cellular metabolism in T cell development, activation, differentiation, and function during NASH. Additional studies are also needed to clarify if T cell activation in the liver during NASH is antigen-dependent and the identity of the antigenic stimuli. As changes in the microbiota can influence intestinal T cells through innate immune mechanisms (124), an interesting possibility is that antigens translocated from a gut into the liver due to increased intestinal permeability can directly stimulate T cell responses during NASH.

Current experimental approaches that target entire T cell subsets such as total CD4 and CD8 depletions do not take into consideration the heterogeneity of these cells. The use of single-cell technologies such as scRNA-seq and mass cytometry offers greater insight into the role of specific T cell populations that will help target NASH-associated pathogenic cells with more specificity. Future studies are also expected to determine whether T cells interact with other liver immune cells such as B cells and macrophages, as well as non-immune cells including hepatocytes and HSCs. For example, the pro-fibrotic or anti-fibrotic roles and potential mechanisms of HSC activation by the different T cell subsets in NASH are poorly understood. As there are important differences between the murine and human immune systems, future studies should account for these variations to help translate preclinical data to the treatment of human disease. Finally, a better understanding of the role of newly discovered T cell subsets and phenotypes, their effector functions, and mechanisms of activation can be exploited for designing future targeted therapies for NASH without compromising the efficacy of T cell homeostatic functions.

## Author Contributions

PH, AB, HW, DP, and XR wrote the manuscript. All authors critically revised the manuscript. All authors contributed to the article and approved the submitted version.

## Funding

PH received support from the AASLD Foundation (Pinnacle Research Award) and National Institute of Diabetes and Digestive and Kidney Diseases (NIDDK) of the National Institutes of Health (NIH) under the Award Numbers P30DK084567 and R01DK130884. AB is supported by the NIH grant K01DK124358 and the Mayo Clinic Center for Biomedical Discovery Career Development Award. DP received support from the NIH grants P30DK084567 and 5T32DK007352-42. XR is supported by NIH grants R01DK122056 and R01HL155993, and the American Association of Immunologists Careers in Immunology Award.

## Conflict of Interest

The authors declare that the research was conducted in the absence of any commercial or financial relationships that could be construed as a potential conflict of interest.

## Publisher’s Note

All claims expressed in this article are solely those of the authors and do not necessarily represent those of their affiliated organizations, or those of the publisher, the editors and the reviewers. Any product that may be evaluated in this article, or claim that may be made by its manufacturer, is not guaranteed or endorsed by the publisher.

## Abbreviations

CD-HFD, Choline-deficient high-fat diet; cDC, Classical dendritic cell; DC, Dendritic cell; FOXP3, Forkhead box protein P3; IL, Interleukin; IFN, Interferon; iNKT, Invariant natural killer T cell; HCC, Hepatocellular carcinoma; HSC, Hepatic stellate cell; HFHC, High-fat, high-carbohydrate; KC, Kupffer cell; MAdCAM-1, Mucosal addressin cell adhesion molecule 1; MAIT, Mucosal-associated invariant T; MCD, Methionine- and choline-deficient; MHC, Major histocompatibility complex; MoMF, Monocyte-derived macrophage; NAFL, Nonalcoholic fatty liver; NAFLD, Nonalcoholic fatty liver disease; NASH, Nonalcoholic steatohepatitis; NKT, Natural killer T cell; TCR, T cell receptor; TGF-β, Transforming growth factor beta; Th, T helper; TNF, Tumor necrosing factor; Treg, Regulatory CD4 T cell; TRM, Tissue-resident memory.

## References

[1] Chalasani N Younossi Z Lavine JE Charlton M Cusi K Rinella M. The Diagnosis and Management of Nonalcoholic Fatty Liver Disease: Practice Guidance From the American Association for the Study of Liver Diseases. Hepatology (2018) 67:328–57. doi: 10.1002/hep.29367 28714183

[2] Sanyal AJ. Past, Present and Future Perspectives in Nonalcoholic Fatty Liver Disease. Nat Rev Gastroenterol Hepatol (2019) 16:377–86. doi: 10.1038/s41575-019-0144-8 31024089

[3] Younossi Z Tacke F Arrese M Chander Sharma B Mostafa I Bugianesi E. Global Perspectives on Nonalcoholic Fatty Liver Disease and Nonalcoholic Steatohepatitis. Hepatology (2019) 69:2672–82. doi: 10.1002/hep.30251 30179269

[4] Loomba R Friedman SL Shulman GI. Mechanisms and Disease Consequences of Nonalcoholic Fatty Liver Disease. Cell (2021) 184:2537–64. doi: 10.1016/j.cell.2021.04.015 PMC12168897 33989548

[5] Younes R Bugianesi E. Should We Undertake Surveillance for HCC in Patients With NAFLD? J Hepatol (2018) 68:326–34. doi: 10.1016/j.jhep.2017.10.006 29122695

[6] Fredrickson G Barrow F Dietsche K Parthiban P Khan S Robert S. Exercise of High Intensity Ameliorates Hepatic Inflammation and the Progression of NASH. Mol Metab (2021) 53:101270. doi: 10.1016/j.molmet.2021.101270 34118476 PMC8255932

[7] Vuppalanchi R Noureddin M Alkhouri N Sanyal AJ. Therapeutic Pipeline in Nonalcoholic Steatohepatitis. Nat Rev Gastroenterol Hepatol (2021) 18:373–92. doi: 10.1038/s41575-020-00408-y 33568794

[8] Parthasarathy G Revelo X Malhi H. Pathogenesis of Nonalcoholic Steatohepatitis: An Overview. Hepatol Commun (2020) 4:478–92. doi: 10.1002/hep4.1479 PMC7109346 32258944

[9] Brandl K Kumar V Eckmann L. Gut-Liver Axis at the Frontier of Host-Microbial Interactions. Am J Physiol Gastrointest Liver Physiol (2017) 312:G413–9. doi: 10.1152/ajpgi.00361.2016 PMC5451561 28232456

[10] Cai J Zhang XJ Li H. The Role of Innate Immune Cells in Nonalcoholic Steatohepatitis. Hepatology (2019) 70:1026–37. doi: 10.1002/hep.30506 30653691

[11] Kazankov K Jorgensen SMD Thomsen KL Moller HJ Vilstrup H George J. The Role of Macrophages in Nonalcoholic Fatty Liver Disease and Nonalcoholic Steatohepatitis. Nat Rev Gastroenterol Hepatol (2019) 16:145–59. doi: 10.1038/s41575-018-0082-x 30482910

[12] Gomez Perdiguero E Klapproth K Schulz C Busch K Azzoni E Crozet L. Tissue-Resident Macrophages Originate From Yolk-Sac-Derived Erythro-Myeloid Progenitors. Nature (2015) 518:547–51. doi: 10.1038/nature13989 PMC5997177 25470051

[13] Bonnardel J T’Jonck W Gaublomme D Browaeys R Scott CL Martens L. Stellate Cells, Hepatocytes, and Endothelial Cells Imprint the Kupffer Cell Identity on Monocytes Colonizing the Liver Macrophage Niche. Immunity (2019) 51:638–54.e639. doi: 10.1016/j.immuni.2019.08.017 31561945 PMC6876284

[14] Imajo K Fujita K Yoneda M Nozaki Y Ogawa Y Shinohara Y. Hyperresponsivity to Low-Dose Endotoxin During Progression to Nonalcoholic Steatohepatitis Is Regulated by Leptin-Mediated Signaling. Cell Metab (2012) 16:44–54. doi: 10.1016/j.cmet.2012.05.012 22768838

[15] Pan J Ou Z Cai C Li P Gong J Ruan XZ. Fatty Acid Activates NLRP3 Inflammasomes in Mouse Kupffer Cells Through Mitochondrial DNA Release. Cell Immunol (2018) 332:111–20. doi: 10.1016/j.cellimm.2018.08.006 30103942

[16] Baeck C Wehr A Karlmark KR Heymann F Vucur M Gassler N. Pharmacological Inhibition of the Chemokine CCL2 (MCP-1) Diminishes Liver Macrophage Infiltration and Steatohepatitis in Chronic Hepatic Injury. Gut (2012) 61:416–26. doi: 10.1136/gutjnl-2011-300304 21813474

[17] Daemen S Gainullina A Kalugotla G He L Chan MM Beals JW. Dynamic Shifts in the Composition of Resident and Recruited Macrophages Influence Tissue Remodeling in NASH. Cell Rep (2021) 34:108626. doi: 10.1016/j.celrep.2020.108626 33440159 PMC7877246

[18] Tran S Baba I Poupel L Dussaud S Moreau M Gelineau A. Impaired Kupffer Cell Self-Renewal Alters the Liver Response to Lipid Overload During Non-Alcoholic Steatohepatitis. Immunity (2020) 53:627–40.e625. doi: 10.1016/j.immuni.2020.06.003 32562600

[19] Hou J Zhang J Cui P Zhou Y Liu C Wu X. TREM2 Sustains Macrophage-Hepatocyte Metabolic Coordination in Nonalcoholic Fatty Liver Disease and Sepsis. J Clin Invest (2021) 131:e135197. doi: 10.1172/JCI135197 PMC7880419 33586673

[20] David BA Rezende RM Antunes MM Santos MM Freitas Lopes MA Diniz AB. Combination of Mass Cytometry and Imaging Analysis Reveals Origin, Location, and Functional Repopulation of Liver Myeloid Cells in Mice. Gastroenterology (2016) 151:1176–91. doi: 10.1053/j.gastro.2016.08.024 27569723

[21] Revelo XS Ghazarian M Chng MH Luck H Kim JH Zeng K. Nucleic Acid-Targeting Pathways Promote Inflammation in Obesity-Related Insulin Resistance. Cell Rep (2016) 16:717–30. doi: 10.1016/j.celrep.2016.06.024 PMC6354586 27373163

[22] Henning JR Graffeo CS Rehman A Fallon NC Zambirinis CP Ochi A. Dendritic Cells Limit Fibroinflammatory Injury in Nonalcoholic Steatohepatitis in Mice. Hepatology (2013) 58:589–602. doi: 10.1002/hep.26267 23322710 PMC3638069

[23] Heier EC Meier A Julich-Haertel H Djudjaj S Rau M Tschernig T. Murine CD103(+) Dendritic Cells Protect Against Steatosis Progression Towards Steatohepatitis. J Hepatol (2017) 66:1241–50. doi: 10.1016/j.jhep.2017.01.008 28108233

[24] Deczkowska A David E Ramadori P Pfister D Safran M At The B. XCR1(+) Type 1 Conventional Dendritic Cells Drive Liver Pathology in non-Alcoholic Steatohepatitis. Nat Med (2021) 27:1043–54. doi: 10.1038/s41591-021-01344-3 34017133

[25] Chen J Liang B Bian D Luo Y Yang J Li Z. Knockout of Neutrophil Elastase Protects Against Western Diet Induced Nonalcoholic Steatohepatitis in Mice by Regulating Hepatic Ceramides Metabolism. Biochem Biophys Res Commun (2019) 518:691–7. doi: 10.1016/j.bbrc.2019.08.111 31472960

[26] Rensen SS Bieghs V Xanthoulea S Arfianti E Bakker JA Shiri-Sverdlov R. Neutrophil-Derived Myeloperoxidase Aggravates non-Alcoholic Steatohepatitis in Low-Density Lipoprotein Receptor-Deficient Mice. PloS One (2012) 7:e52411. doi: 10.1371/journal.pone.0052411 23285030 PMC3527496

[27] Sutti S Albano E. Adaptive Immunity: An Emerging Player in the Progression of NAFLD. Nat Rev Gastroenterol Hepatol (2020) 17:81–92. doi: 10.1038/s41575-019-0210-2 31605031 PMC7222953

[28] Barrow F Khan S Wang H Revelo XS. The Emerging Role of B Cells in the Pathogenesis of NAFLD. Hepatology (2021) 74:2277–86. doi: 10.1002/hep.31889 PMC8463421 33961302

[29] Bruzzi S Sutti S Giudici G Burlone ME Ramavath NN Toscani A. B2-Lymphocyte Responses to Oxidative Stress-Derived Antigens Contribute to the Evolution of Nonalcoholic Fatty Liver Disease (NAFLD). Free Radic Biol Med (2018) 124:249–59. doi: 10.1016/j.freeradbiomed.2018.06.015 29920340

[30] Barrow F Khan S Fredrickson G Wang H Dietsche K Parthiban P. Microbiota-Driven Activation of Intrahepatic B Cells Aggravates NASH Through Innate and Adaptive Signaling. Hepatology (2021) 74:704–22. doi: 10.1002/hep.31755 PMC8377092 33609303

[31] Hosokawa H Rothenberg EV. How Transcription Factors Drive Choice of the T Cell Fate. Nat Rev Immunol (2021) 21:162–76. doi: 10.1038/s41577-020-00426-6 PMC7933071 32918063

[32] Seder RA Ahmed R. Similarities and Differences in CD4+ and CD8+ Effector and Memory T Cell Generation. Nat Immunol (2003) 4:835–42. doi: 10.1038/ni969 12942084

[33] Masopust D Vezys V Marzo AL Lefrancois L. Preferential Localization of Effector Memory Cells in Nonlymphoid Tissue. Science (2001) 291:2413–7. doi: 10.1126/science.1058867 11264538

[34] Germain RN. T-Cell Development and the CD4-CD8 Lineage Decision. Nat Rev Immunol (2002) 2:309–22. doi: 10.1038/nri798 12033737

[35] Kumar BV Connors TJ Farber DL. Human T Cell Development, Localization, and Function Throughout Life. Immunity (2018) 48:202–13. doi: 10.1016/j.immuni.2018.01.007 PMC5826622 29466753

[36] Saravia J Chapman NM Chi H. Helper T Cell Differentiation. Cell Mol Immunol (2019) 16:634–43. doi: 10.1038/s41423-019-0220-6 PMC6804569 30867582

[37] Bennett CL Christie J Ramsdell F Brunkow ME Ferguson PJ Whitesell L. The Immune Dysregulation, Polyendocrinopathy, Enteropathy, X-Linked Syndrome (IPEX) is Caused by Mutations of FOXP3. Nat Genet (2001) 27:20–1. doi: 10.1038/83713 11137993

[38] Kim JM Rasmussen JP Rudensky AY. Regulatory T Cells Prevent Catastrophic Autoimmunity Throughout the Lifespan of Mice. Nat Immunol (2007) 8:191–7. doi: 10.1038/ni1428 17136045

[39] Lee YJ Wang H Starrett GJ Phuong V Jameson SC Hogquist KA. Tissue-Specific Distribution of iNKT Cells Impacts Their Cytokine Response. Immunity (2015) 43:566–78. doi: 10.1016/j.immuni.2015.06.025 PMC4575275 26362265

[40] Lee YJ Holzapfel KL Zhu J Jameson SC Hogquist KA. Steady-State Production of IL-4 Modulates Immunity in Mouse Strains and Is Determined by Lineage Diversity of iNKT Cells. Nat Immunol (2013) 14:1146–54. doi: 10.1038/ni.2731 PMC3824254 24097110

[41] Ribot JC Lopes N Silva-Santos B. Gammadelta T Cells in Tissue Physiology and Surveillance. Nat Rev Immunol (2021) 21:221–32. doi: 10.1038/s41577-020-00452-4 33057185

[42] MacParland SA Liu JC Ma XZ Innes BT Bartczak AM Gage BK. Single Cell RNA Sequencing of Human Liver Reveals Distinct Intrahepatic Macrophage Populations. Nat Commun (2018) 9:4383. doi: 10.1038/s41467-018-06318-7 30348985 PMC6197289

[43] Crispe IN. Hepatic T Cells and Liver Tolerance. Nat Rev Immunol (2003) 3:51–62. doi: 10.1038/nri981 12511875

[44] Heydtmann M Hardie D Shields PL Faint J Buckley CD Campbell JJ. Detailed Analysis of Intrahepatic CD8 T Cells in the Normal and Hepatitis C-Infected Liver Reveals Differences in Specific Populations of Memory Cells With Distinct Homing Phenotypes. J Immunol (2006) 177:729–38. doi: 10.4049/jimmunol.177.1.729 16785572

[45] Dudek M Pfister D Donakonda S Filpe P Schneider A Laschinger M. Auto-Aggressive CXCR6(+) CD8 T Cells Cause Liver Immune Pathology in NASH. Nature (2021) 592:444–9. doi: 10.1038/s41586-021-03233-8 33762736

[46] Wolf MJ Adili A Piotrowitz K Abdullah Z Boege Y Stemmer K. Metabolic Activation of Intrahepatic CD8+ T Cells and NKT Cells Causes Nonalcoholic Steatohepatitis and Liver Cancer *via* Cross-Talk With Hepatocytes. Cancer Cell (2014) 26:549–64. doi: 10.1016/j.ccell.2014.09.003 25314080

[47] Breuer DA Pacheco MC Washington MK Montgomery SA Hasty AH Kennedy AJ. CD8(+) T Cells Regulate Liver Injury in Obesity-Related Nonalcoholic Fatty Liver Disease. Am J Physiol Gastrointest Liver Physiol (2020) 318:G211–24. doi: 10.1152/ajpgi.00040.2019 PMC7052570 31709830

[48] Pfister D Nunez NG Pinyol R Govaere O Pinter M Szydlowska M. NASH Limits Anti-Tumour Surveillance in Immunotherapy-Treated HCC. Nature (2021) 592:450–6. doi: 10.1038/s41586-021-03362-0 PMC8046670 33762733

[49] Bhattacharjee J Kirby M Softic S Miles L Salazar-Gonzalez RM Shivakumar P. Hepatic Natural Killer T-Cell and CD8+ T-Cell Signatures in Mice With Nonalcoholic Steatohepatitis. Hepatol Commun (2017) 1:299–310. doi: 10.1002/hep4.1041 29152605 PMC5687094

[50] Haas JT Vonghia L Mogilenko DA Verrijken A Molendi-Coste O Fleury S. Transcriptional Network Analysis Implicates Altered Hepatic Immune Function in NASH Development and Resolution. Nat Metab (2019) 1:604–14. doi: 10.1038/s42255-019-0076-1 PMC6837876 31701087

[51] Van Herck MA Vonghia L Kwanten WJ Jule Y Vanwolleghem T Ebo DG. Diet Reversal and Immune Modulation Show Key Role for Liver and Adipose Tissue T Cells in Murine Nonalcoholic Steatohepatitis. Cell Mol Gastroenterol Hepatol (2020) 10:467–90. doi: 10.1016/j.jcmgh.2020.04.010 PMC7365964 32360637

[52] Ghazarian M Revelo XS Nohr MK Luck H Zeng K Lei H. Type I Interferon Responses Drive Intrahepatic T Cells to Promote Metabolic Syndrome. Sci Immunol (2017) 2:eaai7616. doi: 10.1126/sciimmunol.aai7616 28567448 PMC5447456

[53] Wang T Sun G Wang Y Li S Zhao X Zhang C. The Immunoregulatory Effects of CD8 T-Cell-Derived Perforin on Diet-Induced Nonalcoholic Steatohepatitis. FASEB J (2019) 33:8490–503. doi: 10.1096/fj.201802534RR 30951375

[54] Koda Y Teratani T Chu PS Hagihara Y Mikami Y Harada Y. CD8(+) Tissue-Resident Memory T Cells Promote Liver Fibrosis Resolution by Inducing Apoptosis of Hepatic Stellate Cells. Nat Commun (2021) 12:4474. doi: 10.1038/s41467-021-24734-0 34294714 PMC8298513

[55] Geginat J Paroni M Maglie S Alfen JS Kastirr I Gruarin P. Plasticity of Human CD4 T Cell Subsets. Front Immunol (2014) 5:630. doi: 10.3389/fimmu.2014.00630 25566245 PMC4267263

[56] Ruterbusch M Pruner KB Shehata L Pepper M. *In Vivo* CD4(+) T Cell Differentiation and Function: Revisiting the Th1/Th2 Paradigm. Annu Rev Immunol (2020) 38:705–25. doi: 10.1146/annurev-immunol-103019-085803 32340571

[57] Zhou L Ivanov II Spolski R Min R Shenderov K Egawa T. IL-6 Programs T(H)-17 Cell Differentiation by Promoting Sequential Engagement of the IL-21 and IL-23 Pathways. Nat Immunol (2007) 8:967–74. doi: 10.1038/ni1488 17581537

[58] Miossec P Kolls JK. Targeting IL-17 and TH17 Cells in Chronic Inflammation. Nat Rev Drug Discov (2012) 11:763–76. doi: 10.1038/nrd3794 23023676

[59] Ziegler SF. FOXP3: Of Mice and Men. Annu Rev Immunol (2006) 24:209–26. doi: 10.1146/annurev.immunol.24.021605.090547 16551248

[60] Gagliani N Amezcua Vesely MC Iseppon A Brockmann L Xu H Palm NW. Th17 Cells Transdifferentiate Into Regulatory T Cells During Resolution of Inflammation. Nature (2015) 523:221–5. doi: 10.1038/nature14452 PMC4498984 25924064

[61] Zhou L Chong MM Littman DR. Plasticity of CD4+ T Cell Lineage Differentiation. Immunity (2009) 30:646–55. doi: 10.1016/j.immuni.2009.05.001 19464987

[62] Levine AG Mendoza A Hemmers S Moltedo B Niec RE Schizas M. Stability and Function of Regulatory T Cells Expressing the Transcription Factor T-Bet. Nature (2017) 546:421–5. doi: 10.1038/nature22360 PMC5482236 28607488

[63] Rau M Schilling AK Meertens J Hering I Weiss J Jurowich C. Progression From Nonalcoholic Fatty Liver to Nonalcoholic Steatohepatitis Is Marked by a Higher Frequency of Th17 Cells in the Liver and an Increased Th17/Resting Regulatory T Cell Ratio in Peripheral Blood and in the Liver. J Immunol (2016) 196:97–105. doi: 10.4049/jimmunol.1501175 26621860

[64] Her Z Tan JHL Lim YS Tan SY Chan XY Tan WWS. CD4(+) T Cells Mediate the Development of Liver Fibrosis in High Fat Diet-Induced NAFLD in Humanized Mice. Front Immunol (2020) 11:580968. doi: 10.3389/fimmu.2020.580968 33013934 PMC7516019

[65] Luo XY Takahara T Kawai K Fujino M Sugiyama T Tsuneyama K. IFN-Gamma Deficiency Attenuates Hepatic Inflammation and Fibrosis in a Steatohepatitis Model Induced by a Methionine- and Choline-Deficient High-Fat Diet. Am J Physiol Gastrointest Liver Physiol (2013) 305:G891–899. doi: 10.1152/ajpgi.00193.2013 24136786

[66] Adams DH Eksteen B. Aberrant Homing of Mucosal T Cells and Extra-Intestinal Manifestations of Inflammatory Bowel Disease. Nat Rev Immunol (2006) 6:244–51. doi: 10.1038/nri1784 16498453

[67] Rai RP Liu Y Iyer SS Liu S Gupta B Desai C. Blocking Integrin Alpha4beta7-Mediated CD4 T Cell Recruitment to the Intestine and Liver Protects Mice From Western Diet-Induced Non-Alcoholic Steatohepatitis. J Hepatol (2020) 73:1013–22. doi: 10.1016/j.jhep.2020.05.047 PMC7839272 32540177

[68] Heinrich B Brown ZJ Diggs LP Vormehr M Ma C Subramanyam V. Steatohepatitis Impairs T-Cell-Directed Immunotherapies Against Liver Tumors in Mice. Gastroenterology (2021) 160:331–45.e336. doi: 10.1053/j.gastro.2020.09.031 33010248 PMC7755834

[69] Ma C Kesarwala AH Eggert T Medina-Echeverz J Kleiner DE Jin P. NAFLD Causes Selective CD4(+) T Lymphocyte Loss and Promotes Hepatocarcinogenesis. Nature (2016) 531:253–7. doi: 10.1038/nature16969 PMC4786464 26934227

[70] Harley IT Stankiewicz TE Giles DA Softic S Flick LM Cappelletti M. IL-17 Signaling Accelerates the Progression of Nonalcoholic Fatty Liver Disease in Mice. Hepatology (2014) 59:1830–9. doi: 10.1002/hep.26746 PMC3975735 24115079

[71] Giles DA Moreno-Fernandez ME Stankiewicz TE Cappelletti M Huppert SS Iwakura Y. Regulation of Inflammation by IL-17A and IL-17f Modulates Non-Alcoholic Fatty Liver Disease Pathogenesis. PloS One (2016) 11:e0149783. doi: 10.1371/journal.pone.0149783 26895034 PMC4760740

[72] Tang Y Bian Z Zhao L Liu Y Liang S Wang Q. Interleukin-17 Exacerbates Hepatic Steatosis and Inflammation in Non-Alcoholic Fatty Liver Disease. Clin Exp Immunol (2011) 166:281–90. doi: 10.1111/j.1365-2249.2011.04471.x PMC3219903 21985374

[73] Ma X Hua J Mohamood AR Hamad AR Ravi R Li Z. A High-Fat Diet and Regulatory T Cells Influence Susceptibility to Endotoxin-Induced Liver Injury. Hepatology (2007) 46:1519–29. doi: 10.1002/hep.21823 17661402

[74] Wang H Zhang H Wang Y Brown ZJ Xia Y Huang Z. Regulatory T Cell and Neutrophil Extracellular Trap Interaction Contributes to Carcinogenesis in Non-Alcoholic Steatohepatitis. J Hepatol (2021). doi: 10.1016/j.jhep.2021.07.032 PMC12888775 34363921

[75] Moreno-Fernandez ME Giles DA Oates JR Chan CC Damen M Doll JR. PKM2-Dependent Metabolic Skewing of Hepatic Th17 Cells Regulates Pathogenesis of Non-Alcoholic Fatty Liver Disease. Cell Metab (2021) 33:1187–204.e1189. doi: 10.1016/j.cmet.2021.04.018 34004162 PMC8237408

[76] Van Herck MA Weyler J Kwanten WJ Dirinck EL De Winter BY Francque SM. The Differential Roles of T Cells in Non-Alcoholic Fatty Liver Disease and Obesity. Front Immunol (2019) 10:82. doi: 10.3389/fimmu.2019.00082 30787925 PMC6372559

[77] Wang H Hogquist KA. How Lipid-Specific T Cells Become Effectors: The Differentiation of iNKT Subsets. Front Immunol (2018) 9:1450. doi: 10.3389/fimmu.2018.01450 29997620 PMC6028555

[78] Hogquist K Georgiev H. Recent Advances in iNKT Cell Development. F1000Res (2020) 9. doi: 10.12688/f1000research.21378.1 PMC7043113 32148771

[79] Wang H Hogquist KA. CCR7 Defines a Precursor for Murine iNKT Cells in Thymus and Periphery. Elife (2018) 7:e34793. doi: 10.7554/eLife.34793 30102153 PMC6115192

[80] Wang H Breed ER Lee YJ Qian LJ Jameson SC Hogquist KA. Myeloid Cells Activate iNKT Cells to Produce IL-4 in the Thymic Medulla. Proc Natl Acad Sci USA (2019) 116:22262–8. doi: 10.1073/pnas.1910412116 PMC6825307 31611396

[81] Crosby CM Kronenberg M. Tissue-Specific Functions of Invariant Natural Killer T Cells. Nat Rev Immunol (2018) 18:559–74. doi: 10.1038/s41577-018-0034-2 PMC6343475 29967365

[82] Mayassi T Barreiro LB Rossjohn J Jabri B. A Multilayered Immune System Through the Lens of Unconventional T Cells. Nature (2021) 595:501–10. doi: 10.1038/s41586-021-03578-0 PMC8514118 34290426

[83] Geissmann F Cameron TO Sidobre S Manlongat N Kronenberg M Briskin MJ. Intravascular Immune Surveillance by CXCR6+ NKT Cells Patrolling Liver Sinusoids. PloS Biol (2005) 3:e113. doi: 10.1371/journal.pbio.0030113 15799695 PMC1073691

[84] Lynch L Nowak M Varghese B Clark J Hogan AE Toxavidis V. Adipose Tissue Invariant NKT Cells Protect Against Diet-Induced Obesity and Metabolic Disorder Through Regulatory Cytokine Production. Immunity (2012) 37:574–87. doi: 10.1016/j.immuni.2012.06.016 PMC4991771 22981538

[85] Guebre-Xabier M Yang S Lin HZ Schwenk R Krzych U Diehl AM. Altered Hepatic Lymphocyte Subpopulations in Obesity-Related Murine Fatty Livers: Potential Mechanism for Sensitization to Liver Damage. Hepatology (2000) 31:633–40. doi: 10.1002/hep.510310313 10706553

[86] Li Z Soloski MJ Diehl AM. Dietary Factors Alter Hepatic Innate Immune System in Mice With Nonalcoholic Fatty Liver Disease. Hepatology (2005) 42:880–5. doi: 10.1002/hep.20826 16175608

[87] Wu L Parekh VV Gabriel CL Bracy DP Marks-Shulman PA Tamboli RA. Activation of Invariant Natural Killer T Cells by Lipid Excess Promotes Tissue Inflammation, Insulin Resistance, and Hepatic Steatosis in Obese Mice. Proc Natl Acad Sci USA (2012) 109:E1143–52. doi: 10.1073/pnas.1200498109 PMC3358828 22493234

[88] Kremer M Thomas E Milton RJ Perry AW van Rooijen N Wheeler MD. Kupffer Cell and Interleukin-12-Dependent Loss of Natural Killer T Cells in Hepatosteatosis. Hepatology (2010) 51:130–41. doi: 10.1002/hep.23292 PMC3761962 20034047

[89] Tang ZH Liang S Potter J Jiang X Mao HQ Li Z. Tim-3/Galectin-9 Regulate the Homeostasis of Hepatic NKT Cells in a Murine Model of Nonalcoholic Fatty Liver Disease. J Immunol (2013) 190:1788–96. doi: 10.4049/jimmunol.1202814 PMC3563933 23296703

[90] Miyagi T Takehara T Uemura A Nishio K Shimizu S Kodama T. Absence of Invariant Natural Killer T Cells Deteriorates Liver Inflammation and Fibrosis in Mice Fed High-Fat Diet. J Gastroenterol (2010) 45:1247–54. doi: 10.1007/s00535-010-0272-y 20596733

[91] Elinav E Pappo O Sklair-Levy M Margalit M Shibolet O Gomori M. Adoptive Transfer of Regulatory NKT Lymphocytes Ameliorates Non-Alcoholic Steatohepatitis and Glucose Intolerance in Ob/Ob Mice and Is Associated With Intrahepatic CD8 Trapping. J Pathol (2006) 209:121–8. doi: 10.1002/path.1950 16482497

[92] Syn WK Oo YH Pereira TA Karaca GF Jung Y Omenetti A. Accumulation of Natural Killer T Cells in Progressive Nonalcoholic Fatty Liver Disease. Hepatology (2010) 51:1998–2007. doi: 10.1002/hep.23599 20512988 PMC2920131

[93] Maricic I Marrero I Eguchi A Nakamura R Johnson CD Dasgupta S. Differential Activation of Hepatic Invariant NKT Cell Subsets Plays a Key Role in Progression of Nonalcoholic Steatohepatitis. J Immunol (2018) 201:3017–35. doi: 10.4049/jimmunol.1800614 PMC6219905 30322964

[94] Borges da Silva H Wang H Qian LJ Hogquist KA Jameson SC. ARTC2.2/P2RX7 Signaling During Cell Isolation Distorts Function and Quantification of Tissue-Resident CD8(+) T Cell and Invariant NKT Subsets. J Immunol (2019) 202:2153–63. doi: 10.4049/jimmunol.1801613 PMC6424602 30777922

[95] Krovi SH Gapin L. Invariant Natural Killer T Cell Subsets-More Than Just Developmental Intermediates. Front Immunol (2018) 9:1393. doi: 10.3389/fimmu.2018.01393 29973936 PMC6019445

[96] Lawand M Dechanet-Merville J Dieu-Nosjean MC. Key Features of Gamma-Delta T-Cell Subsets in Human Diseases and Their Immunotherapeutic Implications. Front Immunol (2017) 8:761. doi: 10.3389/fimmu.2017.00761 28713381 PMC5491929

[97] Chen Y Tian Z. Innate Lymphocytes: Pathogenesis and Therapeutic Targets of Liver Diseases and Cancer. Cell Mol Immunol (2021) 18:57–72. doi: 10.1038/s41423-020-00561-z 33041339 PMC7852564

[98] Li F Hao X Chen Y Bai L Gao X Lian Z. The Microbiota Maintain Homeostasis of Liver-Resident gammadeltaT-17 Cells in a Lipid Antigen/CD1d-Dependent Manner. Nat Commun (2017) 7:13839. doi: 10.1038/ncomms13839 28067223 PMC5227332

[99] Zuniga LA Shen WJ Joyce-Shaikh B Pyatnova EA Richards AG Thom C. IL-17 Regulates Adipogenesis, Glucose Homeostasis, and Obesity. J Immunol (2010) 185:6947–59. doi: 10.4049/jimmunol.1001269 PMC3001125 21037091

[100] Torres-Hernandez A Wang W Nikiforov Y Tejada K Torres L Kalabin A. Gammadelta T Cells Promote Steatohepatitis by Orchestrating Innate and Adaptive Immune Programming. Hepatology (2020) 71:477–94. doi: 10.1002/hep.30952 31529720

[101] Bolte FJ Rehermann B. Mucosal-Associated Invariant T Cells in Chronic Inflammatory Liver Disease. Semin Liver Dis (2018) 38:60–5. doi: 10.1055/s-0037-1621709 PMC6283052 29471566

[102] Diedrich T Kummer S Galante A Drolz A Schlicker V Lohse AW. Characterization of the Immune Cell Landscape of Patients With NAFLD. PloS One (2020) 15:e0230307. doi: 10.1371/journal.pone.0230307 32168345 PMC7069622

[103] Li Y Huang B Jiang X Chen W Zhang J Wei Y. Mucosal-Associated Invariant T Cells Improve Nonalcoholic Fatty Liver Disease Through Regulating Macrophage Polarization. Front Immunol (2018) 9:1994. doi: 10.3389/fimmu.2018.01994 30233587 PMC6131560

[104] Hegde P Weiss E Paradis V Wan J Mabire M Sukriti S. Mucosal-Associated Invariant T Cells Are a Profibrogenic Immune Cell Population in the Liver. Nat Commun (2018) 9:2146. doi: 10.1038/s41467-018-04450-y 29858567 PMC5984626

[105] Bottcher K Rombouts K Saffioti F Roccarina D Rosselli M Hall A. MAIT Cells Are Chronically Activated in Patients With Autoimmune Liver Disease and Promote Profibrogenic Hepatic Stellate Cell Activation. Hepatology (2018) 68:172–86. doi: 10.1002/hep.29782 29328499

[106] Yang JD Hainaut P Gores GJ Amadou A Plymoth A Roberts LR. A Global View of Hepatocellular Carcinoma: Trends, Risk, Prevention and Management. Nat Rev Gastroenterol Hepatol (2019) 16:589–604. doi: 10.1038/s41575-019-0186-y 31439937 PMC6813818

[107] Younossi Z Stepanova M Ong JP Jacobson IM Bugianesi E Duseja A. Nonalcoholic Steatohepatitis Is the Fastest Growing Cause of Hepatocellular Carcinoma in Liver Transplant Candidates. Clin Gastroenterol Hepatol (2019) 17:748–55.e743. doi: 10.1016/j.cgh.2018.05.057 29908364

[108] Huang DQ El-Serag HB Loomba R. Global Epidemiology of NAFLD-Related HCC: Trends, Predictions, Risk Factors and Prevention. Nat Rev Gastroenterol Hepatol (2021) 18:223–38. doi: 10.1038/s41575-020-00381-6 PMC8016738 33349658

[109] Baffy G Brunt EM Caldwell SH. Hepatocellular Carcinoma in Non-Alcoholic Fatty Liver Disease: An Emerging Menace. J Hepatol (2012) 56:1384–91. doi: 10.1016/j.jhep.2011.10.027 22326465

[110] Mann JP Valenti L Scorletti E Byrne CD Nobili V. Nonalcoholic Fatty Liver Disease in Children. Semin Liver Dis (2018) 38:1–13. doi: 10.1055/s-0038-1627456 29471561

[111] Giraud J Chalopin D Blanc JF Saleh M. Hepatocellular Carcinoma Immune Landscape and the Potential of Immunotherapies. Front Immunol (2021) 12:655697. doi: 10.3389/fimmu.2021.655697 33815418 PMC8012774

[112] Pinyol R Torrecilla S Wang H Montironi C Pique-Gili M Torres-Martin M. Molecular Characterisation of Hepatocellular Carcinoma in Patients With non-Alcoholic Steatohepatitis. J Hepatol (2021) 75:865–78. doi: 10.1016/j.jhep.2021.04.049 PMC12164395 33992698

[113] Grohmann M Wiede F Dodd GT Gurzov EN Ooi GJ Butt T. Obesity Drives STAT-1-Dependent NASH and STAT-3-Dependent HCC. Cell (2018) 175:1289–306.e1220. doi: 10.1016/j.cell.2018.09.053 30454647 PMC6242467

[114] Shalapour S Lin XJ Bastian IN Brain J Burt AD Aksenov AA. Inflammation-Induced IgA+ Cells Dismantle Anti-Liver Cancer Immunity. Nature (2017) 551:340–5. doi: 10.1038/nature24302 PMC5884449 29144460

[115] Hirsova P Bohm F Dohnalkova E Nozickova B Heikenwalder M Gores GJ. Hepatocyte Apoptosis Is Tumor Promoting in Murine Nonalcoholic Steatohepatitis. Cell Death Dis (2020) 11:80. doi: 10.1038/s41419-020-2283-9 32015322 PMC6997423

[116] Gomes AL Teijeiro A Buren S Tummala KS Yilmaz M Waisman A. Metabolic Inflammation-Associated IL-17a Causes Non-Alcoholic Steatohepatitis and Hepatocellular Carcinoma. Cancer Cell (2016) 30:161–75. doi: 10.1016/j.ccell.2016.05.020 27411590

[117] Hirsova P Gores GJ. Fatty Liver Progression and Carcinogenesis: Beware of Pathogenic T Cells. Med (2021) 2:453–5. doi: 10.1016/j.medj.2021.04.020 35590223

[118] Xu J Ma HY Liu X Rosenthal S Baglieri J McCubbin R. Blockade of IL-17 Signaling Reverses Alcohol-Induced Liver Injury and Excessive Alcohol Drinking in Mice. JCI Insight (2020) 5:e131277. doi: 10.1172/jci.insight.131277 PMC7098802 32051339

[119] Burnstock G. Purinergic Signalling: Therapeutic Developments. Front Pharmacol (2017) 8:661. doi: 10.3389/fphar.2017.00661 28993732 PMC5622197

[120] Barber DL Wherry EJ Masopust D Zhu B Allison JP Sharpe AH. Restoring Function in Exhausted CD8 T Cells During Chronic Viral Infection. Nature (2006) 439:682–7. doi: 10.1038/nature04444 16382236

[121] Raffin C Vo LT Bluestone JA. Treg Cell-Based Therapies: Challenges and Perspectives. Nat Rev Immunol (2020) 20:158–72. doi: 10.1038/s41577-019-0232-6 PMC7814338 31811270

[122] Ibrahim SH Hirsova P Malhi H Gores GJ. Animal Models of Nonalcoholic Steatohepatitis: Eat, Delete, and Inflame. Dig Dis Sci (2016) 61:1325–36. doi: 10.1007/s10620-015-3977-1 PMC4838538 26626909

[123] Schuster S Cabrera D Arrese M Feldstein AE. Triggering and Resolution of Inflammation in NASH. Nat Rev Gastroenterol Hepatol (2018) 15:349–64. doi: 10.1038/s41575-018-0009-6 29740166

[124] Kubinak JL Petersen C Stephens WZ Soto R Bake E O’Connell RM. MyD88 Signaling in T Cells Directs IgA-Mediated Control of the Microbiota to Promote Health. Cell Host Microbe (2015) 17:153–63. doi: 10.1016/j.chom.2014.12.009 PMC4451207 25620548

